# Different Scripts, Different Casts: A Crime Script Analysis Indicating Intimate Partner Violence Is Not All the Same

**DOI:** 10.1177/10778012231153361

**Published:** 2023-02-12

**Authors:** Christine T. Carney, Mark R. Kebbell, Li Eriksson, Regan M. Carr

**Affiliations:** 1 Griffith University, Griffith Criminology Institute, Mt Gravatt, Queensland, Australia; 2 1969 Queensland University of Technology, School of Justice, Brisbane, Queensland, Australia

**Keywords:** crime scripts, intimate partner violence, typology, cluster analysis, violence against women

## Abstract

Crime script analysis was used to analyze intimate partner violence diversity by identifying variables significantly associated with different script tracks. Qualitative thematic analysis using official police administrative data from Queensland, Australia, was used to develop an intimate partner violence protoscript (*n* = 40), followed by quantitative hierarchical cluster analysis and cross-tabulations to examine diversity within scripts. Four diverse script tracks were identified: “escalating jealousy,” “persistently possessive,” “controlling victim agency,” and “enduring argument.” Intimate partner diversity exists with divisions based on statistically significant variables. Implications for situational crime prevention and the use of mixed methods for strengthening crime script analysis are discussed.

With one in three women experiencing physical or sexual violence World Health Organization, 2021b) and approximately 38.0% of murdered women dying at the hands of a male intimate partner (World Health Organization, 2021a), the need to understand how and why this form of violence occurs is great. One method for identifying and understanding the intricacies of intimate partner violence is crime script analysis. Crime script analysis refers to identifying the sequence of events in the lead up to, during, and after a crime event (Cornish, 1994a; Leclerc & Reynald, 2016). This includes factors relevant to target selection (need, opportunity), preparation (obtaining tools), entry to setting (residence, business, vehicle), doing (commit crime), and exit from scene (Cornish, 1994a). A crime script approach also enables differing levels of analysis from the broad (protoscript) to the specific (script track) (refer to Table 1) (Dehghanniri & Borrion, 2021; Ekblom & Gill, 2016). Originally developed to better understand auto-theft, this method has proven useful in understanding interpersonal crimes such as sexual offending (Beauregard et al., 2007; Leclerc et al., 2011) as well as general crimes (Hutching & Holt, 2015; Kennedy et al., 2018; Lord et al., 2017). This is because crime scripts can provide a visual representation of the stages of crime commission as well as points for prevention and intervention (Cornish, 1994a; Dehghaniri & Borrion, 2021; Leclerc & Reynald, 2016). The benefits of understanding where offenders and victims are likely to come into contact and how offenders select their targets, close in, and act out criminal behaviors offer police and other agencies greater understanding of the context in which crime occurs. Extending analysis to incorporate victim responses can further enhance understanding of this form of abuse and support appropriate intervention.

**Table 1.** Crime Script Levels of Analysis.

Level of abstraction	Label	Description	Example
Generic	Universal	Generic	Any offense
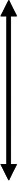	Metascript	Offense type	Interpersonal violence
	Protoscript	Offense subgroup	Intimate partner violence
	Script	Specific offense characteristics	Male-perpetrated intimate partner violence against female victims
Specific	Track	Specific circumstances of specific offences	Intimate partner violence between ex-de facto male offender and female victim in the victims’ residence

*Note*. Borrion (2013), Cornish (1994a), and Leclerc et al. (2011).

## Knowledge Gaps and Study Aims

The current study applies a crime script approach to understanding intimate partner violence. Crime script analysis is one way researchers and practitioners can better understand the stages within a crime event and use this knowledge to develop preventative measures to disrupt its recurrence (Cornish, 1994b). The current study extends past research examining the crime commission process by focusing on both offender action and victim response. It acknowledges that not all incidents of intimate partner violence are the same. As identified in several studies, diversity within intimate partner violence exists (e.g., M. P. Johnson, 2008; Holtzworth-Munroe et al., 2000). While crime script research has focused on identifying one overarching “protoscript,” greater specificity may be required to understand the diversity within crime scripts, especially for offences such as intimate partner violence.

In the current paper, we extend past research in two main ways. First, we analyze both offender actions and victim responses based on police administrative data to develop an intimate partner violence protoscript. This includes analyzing risk items from the Domestic Violence – Protective Assessment Framework (DV-PAF); a validated risk assessment used by police to support decision making at the time of a call for service that incorporates 22 evidence-based risk items (Kebbell, 2019; Queensland Police Service, 2019). Second, we explore diversity *within* crime scripts (so-called “script tracks”) by running cluster analysis. We use the term “intimate partner violence” to refer to violence between current and former intimate partners. Such violence may consist of physical, sexual, psychological, emotional, or financial abuse or coercive behaviors designed to intimidate, threaten, or control a significant other (Douglas, 2019; Krigel & Benjamin, 2020; World Health Organization, 2013). We use the term “offender” to refer to the primary person using violence and the term “victim^1^” to refer to the person experiencing violence (often referred to as “respondent” and “aggrieved” within policing/legal contexts). The key aims of the study are to: (a) apply crime script analysis to intimate partner violence, (b) examine offender–victim interactions, (c) explore diversity across script tracks, and (d) identify variables significantly associated with different script tracks.

## Literature Review

Previous use of crime script analysis in establishing the overarching process of intimate partner violence is limited (Boxall et al., 2018). To date, only a single study has explicitly used crime script analysis in this way (Boxall et al., 2018). Based on data from an Australian policing jurisdiction, the study examined first time domestic violence cases over a 12-month period through an in-depth analysis of 100 randomly selected cases and a final sample of 50 cases (Boxall et al., 2018). Crime classifications included assault, threats, stalking, and breach of domestic violence orders (Boxall et al., 2018). Analysis of cases involved the review of police narratives; victim, offender, and witness statements; and official records involving a male offender and a female victim (Boxall et al., 2018). A protoscript of domestic violence was then developed based upon an existing child sexual offending script developed by Leclerc et al. (2011) (Boxall et al., 2018). The study showed crime script analysis could be used effectively to understand intimate partner violence at the broad level of analysis and the potential for further exploration of intimate partner violence in greater levels of detail. While informative, a limitation of the study was that it focused on violence toward the victim as a key stage of the script (Boxall et al., 2018). Such an approach fails to account for interactions between offenders and victims and how these interactions influence a continuation or cessation of violence. Indeed, studies emphasize the importance of understanding victim and offender interactions when examining crime events (Fossi et al., 2005; Leclerc et al., 2011; Luckenbill, 1977). For example, an offender-focused approach fails to consider the various methods used by victims to de-escalate the situation, protect children who may be present, and/or protect themselves (see, e.g., Meyer, 2010).

Although a few crime scripts studies to date have explored offender–victim interactions (Leclerc et al., 2011), several studies have explored variations in victimization and offending and offender typologies using multiple correspondence analysis (González-Álvarez et al., 2021), cluster analysis (Dawson & Piscitelli, 2021; Mennicke, 2019), and behavior sequence analysis (BSA; Keatley et al., 2021) (Quinn-Evans et al., 2021). In one study (Mennicke, 2019) cluster analysis was performed using quantitative cross-sectional survey data (*n* = 714) to group “like” observations together. Differences between clusters were then identified statistically with each case categorized and mapped onto M. P. Johnson's (2008) typologies—intimate terrorist, violent resistance, situational couple violence, or mutual violent control (Mennicke, 2019). Benefits of this approach include an expanded understanding of intimate partner violence, identifying victim responses to abuse, and raising awareness of the range of violence experienced (Mennicke, 2019). Another study explored combinations of risk factors evident within intimate partner homicides using a Canadian Death Review data sample (Dawson & Piscitelli, 2021). Using cluster analysis, the study found significant differences between clusters based on depression and relationship status (Dawson & Piscitelli, 2021).

 BSA has been used in a comparable way to crime scripts to develop temporal accounts of interpersonal violence with samples of 30 to 60 cases (Keatley et al., 2021; Quinn-Evans et al., 2021). Using similar principles to crime script analysis, BSA maps events and behaviors over time (Keatley et al., 2021). Behaviors are coded and categorized qualitatively (Keatley et al., 2021) in the same way crime scripts are developed to better understand the step-by-step nature of crime (Leclerc et al., 2011). The sequences identified are then compared statistically (Keatley et al., 2021; Quinn-Evans et al., 2021). Benefits of the BSA method include the ability to chart both physical and non-physical forms of abuse, identify points of transition from one behavior to another, and provide a visual graphic of the complex nature of intimate partner violence. Drawing on this methodology, the current study builds upon this aspect of BSA to strengthen the crime script approach. This study aims to show how administrative data, including victim, offender, and witness accounts, can be used to build greater understanding of the procedural aspects of intimate partner violence to better inform prevention.

### Crime Script Analysis: Theory and Method

Born from an exploration of how humans process knowledge (Schank & Abelson, 1977) and the connection between these processes and the commission of crime (Cornish, 1994a), crime script analysis offers insight into how knowledge structures inform behavior, routines, and action (Hewstone, 1989). It does this by providing a framework for understanding each stage of the decision-making process used within specific contexts, from target selection through to exiting the scene (Leclerc, 2014). Understanding these aspects of crime, particularly in terms of intimate partner violence, is vital in reducing the considerable harm caused by this form of violence. In his seminal work, Cornish (1994a) developed the first explicit crime script based on “ringing” (a form of auto theft), which included five key elements: theft of a vehicle, concealment, disguise, sales market, and disposal. This depiction of the procedural aspects of crime highlighted the inter-dependencies within and potential variants across each element of the crime commission process by mapping out the individual and alternative steps at each stage of the process (Cornish, 1994a). Previous methods of analyzing crime had not offered the opportunities found within crime script analysis to understand crime commission at differing levels of specificity. While auto-theft and intimate partner violence are quite different crimes, the value of this approach lies in the utility of scripts to provide broad and detailed levels of analyzes for any crime type.

The greatest level of specificity is found in so-called script tracks, where characteristics of certain crimes (e.g., intimate partner violence) are analyzed in detail. Specificity allows for the identification of prevention and intervention opportunities within crime types and can be used to guide the development of policy and practice. Specificity also enables tailored approaches at the individual level, which is useful for frontline police. The use of this methodology to better understand intimate partner violence has been limited to date, but the potential is significant.

Two separate (but related) theories are relevant to crime scripts and the current study. First, the rational choice perspective, developed with deterrence and situational crime prevention in mind (Akers, 1990; Cornish & Clarke, 2014), adds to the strength of crime script analysis by explaining the decision-making processes involved in crime (Akers, 1990; Leclerc et al., 2011). Drawing on principles of economics, rational choice assists in understanding the costs/benefits of committing crime (Akers, 1990). Crime script studies have drawn upon the rational choice perspective to explain offender decision making in terms of target selection, modus operandi, and timing of crime events (Akers, 1990; Clarke & Harris, 1992; Cornish & Clarke, 2008; Wortley, 2011). Some studies have also explored the role of rational choice from the victims’ perspective, including how victims respond to the actions of offenders to minimize harm (Leclerc et al., 2011). It has been argued that rational choice cannot be used for understanding interpersonal crimes given the perception that these crimes are expressive or reactive (Hayward, 2007). Others would suggest that this is not the case (Farrell, 2010) and that for particularly coercive or controlling relationships, violence is often planned (Stark, 2009). Indeed, the most frequently used violence risk assessment tool, the HCR-20, analyzes offender decision making with the concept of motivators, disinhibitors, and destabilizers, to predict future violence (Douglas & Reeves, 2011). This has a great deal of empirical support. Victim studies have also discussed rational decision making in deciding whether to stay in violent relationships (Meyer, 2012).

Another theory of relevance to crime scripts and the current study is social interactionist theory because of how it considers the situational and interpersonal factors evident in crime events and how victims and offenders interact (Felson & Tedeschi, 1993; Leclerc et al., 2011). This is of particular interest to the development of intimate partner violence crime scripts. Offender–victim interactions can provide greater understanding of decision-making processes evident within each stage of an intimate partner violence incident and can support tailored situational crime prevention measures (Felson & Tedeschi, 1993; Leclerc et al., 2011). As discussed by Leclerc et al. (2011) in his analysis of child sex offender scripts, the way victims respond to an offender can influence crime outcomes.

### Intimate Partner Violence

A range of terms are used to describe relational violence such as intimate partner violence (Satyen et al., 2018), domestic violence (Dowling et al., 2021), domestic abuse (Irving-Clarke et al., 2021), and wife battering (Areh et al., 2020). In this paper, the term intimate partner violence is used to refer to violence committed by a current or former intimate partner. This includes acts that are physically, sexually, emotionally, psychologically, or economically abusive, threatening, intimidating or coercive and controlling (and often) experienced as an ongoing pattern of abuse (Boxall & Morgan, 2021; Stark, 2020b). Despite decades of research exploring a variety of topics associated with intimate partner violence (Kebbell, 2019; McEwan et al., 2019; Myhill & Hohl, 2019), criminal justice related (Nancarrow et al., 2020), coercive control (Myhill, 2015; Stark, 2020a), depression and anxiety orders (Ahmadabadi et al., 2020), a clear understanding of what works to reduce its prevalence is yet to be identified.

Typologies of intimate partner violence have been developed to support treatment options (Saunders, 1992; Stare & Fernando, 2014), understand differing levels of risk (González-Álvarez et al., 2021; Holtzworth-Munroe & Stuart, 1994; Kivisto, 2015), and support appropriate interventions (Love et al., 2020). The most well-known typologies are those of M. P. Johnson (1995) and Holtzworth-Munroe and Stuart (1994). M. P. Johnson (1995) typology identified two significant forms of intimate partner violence—patriarchal terrorism and common couple violence. Patriarchal terrorism more recently described as intimate terrorism (M. P. Johnson, 2008) is the most pervasive form of violence because of the methods used to coerce, control, intimidate, and threaten a victim. Common couple violence, also referred to as situational couple violence (M. P. Johnson, 2008), may include severe violence but does not involve ongoing coercive or controlling behaviors.

### Applying Crime Script Analysis to Intimate Partner Violence

Though crime scripts have been used to explain a wide variety of violent forms of crime, such as sex crimes and fire setting (e.g., Beauregard et al., 2007; Butler & Gannon, 2015; Chiu, 2015; Davies et al., 2013; Leclerc et al., 2011), their utility for intimate partner violence has rarely been explored (though see a notable exception below). A similar, though slightly different, approach to violent crime is seen in the “situated transactions” framework, which has been used to explain violent crimes such as homicide between strangers and known parties (Luckenbill, 1977), and serial sexual offending (Beauregard et al., 2007). As with crime script analysis, situated transaction theory highlights key stages of victim–offender interactions (Luckenbill, 1977; Pizarro et al., 2019; Savitz et al., 1991). These may include victim actions perceived by the offender as insulting or offensive (Luckenbill, 1977; Savitz et al., 1991) and offender actions designed to “save face” (Luckenbill, 1977). Studies using the situated transaction approach have identified different types of transaction including “establishing the moral order” by way of ensuring the sanctity of relationships or by defending ideals, demanding esteem from others, “saving face,” and protecting status (Pizarro et al., 2019). These classifications have relevance for intimate partner violence within intimate relationships, particularly when exploring differences across incidents based on the presence or absence of variables such as jealousy or coercive control.

While some stages within the situated transaction approach may be useful when examining intimate partner violence, this approach fails to account for historical and situational factors outside of the immediate victim–offender interaction. It also fails to account for multiple events over time and the way in which issues can manifest and influence escalating behaviors. As some scholars have argued, the situated transaction approach fails in its explanation of violence by overemphasizing the mutuality of conflict, lacking distinction between violent and non-violent events and gender differences in offending (Deibert & Miethe, 2003). Although situated transaction may support our understanding of some crime types, crime script analysis extends our understanding of victim–offender interactions by combining historical and situational factors. It also contributes to understanding of the decision-making process of both victims and offenders at various stages of a script and how these decisions influence escalation or de-escalation within a single incident or across multiple events. Crime scripts also provide greater opportunities to identify situational prevention measures and greater levels of specificity.

Despite the potential of crime scripts to assist in preventing intimate partner violence incidents, to date only one published paper has used crime scripts to understand intimate partner violence (Boxall et al., 2018). Based on data from an Australian policing jurisdiction, the study examined a sample of first-time intimate partner violence cases over a 12 month period (*n* = 50) that included physical and non-physical forms of violence (Boxall et al., 2018). The study explored only those cases with a male offender and female victim with the protoscript informed by the child sexual offending script developed by Leclerc et al. (2011). Historical and situational preconditions were included in the protoscript to provide greater context to the incident reviewed and included a history of violence, relationship problems, separation, and substance use (Boxall et al., 2018). Drawing on information from police narratives, victim, offender, and witness statements, and official records, the study found that intimate partner violence incidents follow similar protoscript stages to other crime types, including contact made with victim, conflict, “tipping point,” violence, de-escalation of violence, and end of contact (Boxall et al., 2018).

## Method

### Sample

A University Human Research Ethics Committee and the Queensland Police Service Research Committee granted ethics approval. The study location (Queensland, Australia) has a population of 5.2 million people spread across a diverse 1.7 million km^2^ area (Queensland Government Statistician's Office, 2021). The sample was drawn from police administrative data extracted from the Queensland Police Records and Information Management Exchange (QPRIME), which stores all information on matters referred or attended to by police. Data used for the current study included calls for service for intimate partner resulting in a protection order application, police narratives, witness, victim, and offender statements, and outcomes from the domestic violence protective assessment framework (DV-PAF). The DV-PAF is used by police to identify risks such as threats, separation, pregnancy, strangulation, mental health, level of fear, and perceived risk level (Queensland Police Service, 2019).

The sample was drawn from a larger dataset and randomly selected using an online random integer generator. For this study, the random sample (*n* = 200) was filtered to include only incidents resulting in an application for a protection order linked to a DV-PAF report. It excluded calls for service related to breaches of existing protection orders (*n* = 69).^2^ The final dataset included DV-PAF indicators and police narratives drawn from applications for a protection order. Duplicates were removed (*n* = 3) along with calls for service that involved family members other than intimate partners (*n* = 3). Calls for service were also removed if the attending officer had doubts as to the truthfulness of accounts or insufficient information was available to develop a script (*n* = 21). Two calls for service involving female offenders were also removed due to the low incidence. The final sample comprised of 40 calls for service involving couples aged 18 years and over, residing in Queensland (Australia) at the time the call for service was made to police. Table 2 provides descriptive details about the sample.

**Table 2.** Descriptive Characteristics of Protoscript/Track Sample.

	Cluster 1 Escalating jealousy		Cluster 2 Persistently possessive		Cluster 3 Controlling victim agency		Cluster 4 Enduring argument	
	*n*	%	*n*	%	*n*	%	*n*	%
Victim age								
<25 years	3	7.5	0	0.0	0	0.0	5	12.5
25–34 years	2	5.0	6	15.0	4	10.0	9	22.5
35–49 years	1	2.5	0	0.0	0	0.0	6	15.0
50 years and over	1	2.5	2	5.0	0	0.0	0	0.0
Not stated	0	0.0	0	0.0	1	0.0	0	0.0
Offender age								
<25 years	2	5.0	0	0.0	0	0.0	3	7.5
25–34 years	1	2.5	4	10.0	2	5.0	6	15.0
35–49 years	3	7.5	2	5.0	2	5.0	9	22.5
50 years and over	1	2.5	2	5.0	0	0.0	2	5.0
Not stated	0	0.0	0	0.0	1	2.5	0	0.0
Victim ethnicity								
Aboriginal and/or Torres Strait Islander	0	0.0	0	0.0	0	0.0	5	12.5
Australian (other)	3	7.5	4	10.0	4	10.0	10	25.0
Other	2	5.0	0	0.0	1	2.5	0	0.0
Offender ethnicity								
Aboriginal and/or Torres Strait Islander	0	0.0	0	0.0	0	0.0	5	12.5
Australian (other)	4	10.0	3	7.5	3	7.5	9	22.5
Other	1	2.5	1	2.5	2	5.0	1	2.5
Relationship characteristics								
Current	6	15.0	3	7.5	3	7.5	13	32.5
Former	1	2.5	2	5.0	0	0.0	5	12.5
On/off	0	0.0	3	7.5	2	5.0	1	2.5
Children in relationship	5	12.5	3	7.5	5	12.5	7	17.5
Co-reside	4	10.0	6	15.0	4	10.0	10	25.0
Relationship length								
Less than 12 months	0	0.0	2	7.7	1	3.8	2	7.7
12 months to 5 years	3	11.5	2	7.7	1	3.8	7	23.0
Greater than 5 years	2	7.7	3	11.5	1	3.8	2	7.7

### Coding Process

**Figure 1.**
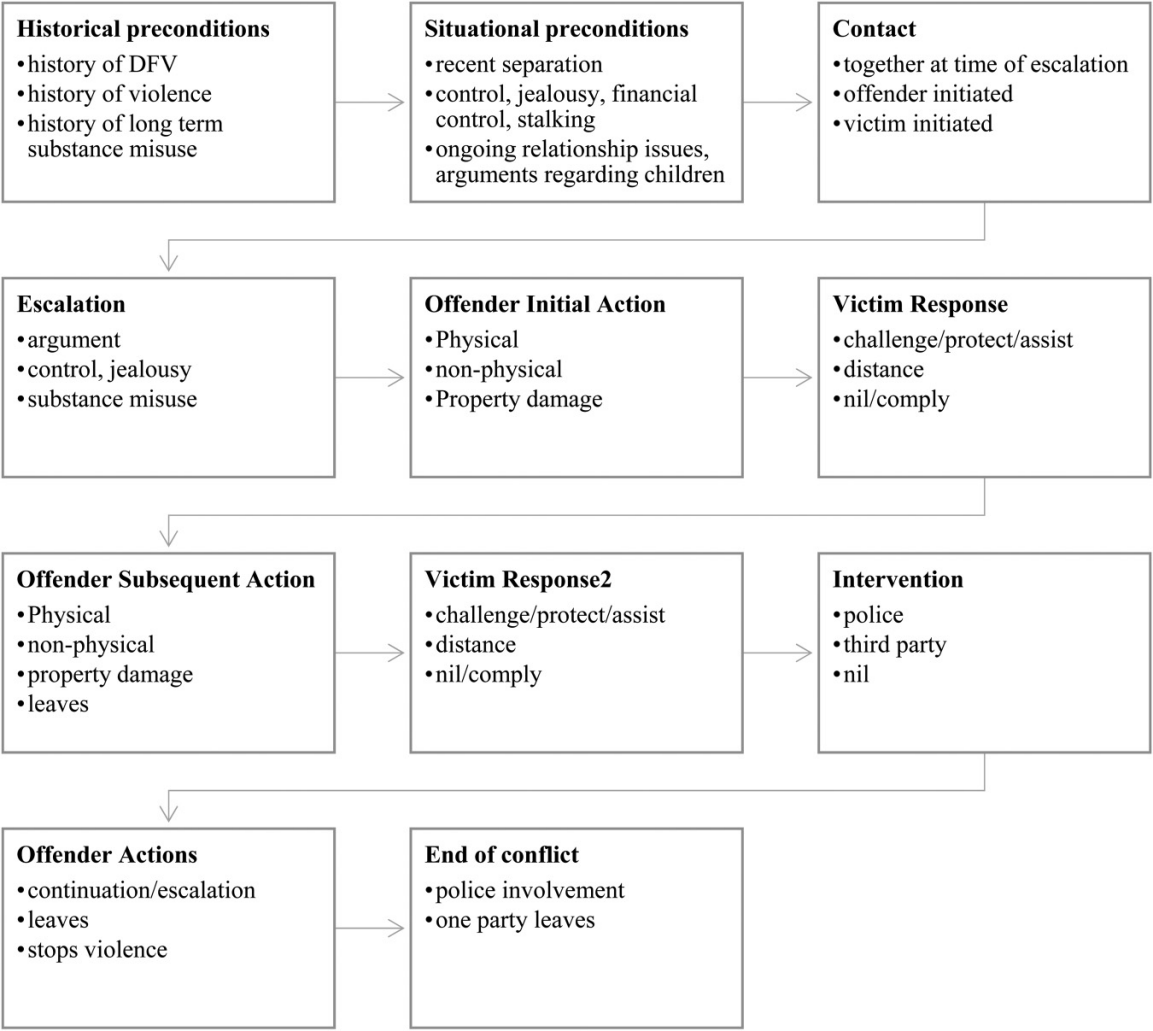
Intimate partner violence protoscript.

As police narratives are free flowing text fields, thematic analysis is suitable for coding as it provides for flexibility and depth in understanding of the content (Braun & Clarke, 2006). Following a multi-step coding process, narratives were analyzed to identify the characteristics relevant to each stage of the crime commission process at the protoscript level. This included identifying factors related to historical preconditions, for example, a history of violence, history of intimate partner violence, or long-term substance misuse. Situational preconditions such as ongoing relationship issues, recent separation, or substance misuse just prior to or during the incident, contact made between offender and victim, offender actions, victim response, intervention, and end of contact/conflict were also included. Narratives were summarized individually, and themes throughout the text were coded by the main researcher, with coding refined over several iterations. To ensure inter-rater reliability, a second person familiar with police administrative data independently coded the sample. The researchers compared the codes, and where coding differed, consensus was reached by both coders coming together and reviewing the information. The information was then mapped onto an existing intimate partner protoscript framework (Boxall et al., 2018; Cornish, 1994a; Leclerc et al., 2011; Figure 1).

#### Measures

A code book was developed from the thematic analysis of incident characteristics aligned with each stage of the crime-commission process to create a range of variables relevant to both the protoscript and track level. These variables (see Figure 1) recorded as present or absent (1,0) included the setting in which the intimate partner violence occurred (private, public, or both), how contact between offender and victim was made (already together at location, offender initiated), escalation based on the main perceived motivation for the violence (argument, control, jealousy), offender actions (physical/non-physical violence), victim response (challenge/distance), intervention (police, third party), and end of contact/conflict (police involvement, one party leaves). Further data used to support greater understanding of the historical and situational preconditions present in the relationship were drawn from police narratives and DV-PAF items and recorded as present or absent (1,0). This information included history of domestic violence, history of general violence, and history of substance misuse, intoxication, or ongoing relationship issues at time of incident or recent financial issues.

To allow for a more nuanced understanding of individual actions at the script track level, subjective variables such as jealousy and control were coded based on key words evident within police narratives. Where the narrative documented arguments over the victim texting/speaking with the opposite sex, accusations of infidelity, possessiveness, and derogatory name calling such as whore, the theme of jealousy was recorded. Calls for service involving the offender threatening violence or self-harm if demands were unmet, forbidding the victim to do something, constantly calling, controlling household finances, stalking, or controlling victim movements were recorded as controlling behavior. Many forms of physical violence were noted in the narratives ranging from minor (pushing) to severe (assault breaking bones). Regardless of severity, all forms were coded as physical violence, except for strangulation, which was coded separately to highlight the heightened level of risk associated with this form of violence. Calls for service involving verbal abuse only (including threats to kill) were coded as non-physical. An additional item—Explicit threats to kill—was included as these explicit threats specifically detailed how the offender planned to carry out the threat (e.g., manner of death, location buried).

#### Analytical Technique

The protoscript was adapted by using the existing protoscript framework described in Leclerc et al. (2011) and Boxall et al. (2018) and adding stages to better highlight the sequence of events evident across incidents (e.g., victim response and offender reaction to interventions). Once the protoscript was developed, further analysis was conducted to explore diversity across the sample using cluster analysis in SPSS (v. 27.0) (Fox & Escue, 2021). In total, 38 variables were analyzed and recorded as present or absent (1,0). Hierarchical cluster analysis was performed using Ward's Linkage method based on squared Euclidean distances. A comparative analysis of three different clustering methods using qualitative data with small sample sizes suggests that the use of hierarchical clustering, K-means, and latent class analysis can produce similar levels of accuracy in samples as small as 50 (Henry et al., 2015, 1007). This study also found that the use of hierarchical clustering could produce *usable solutions with samples of 20* (Henry et al., 2015, p. 1009). Therefore, this method was chosen as it is useful for grouping like cases together and can be used with small sample sizes (Henry et al., 2015). Based on results of the dendrogram produced by hierarchical cluster analysis, a four-cluster solution was considered suitable, with cluster membership saved as a categorical variable within SPSS (Lee et al., 2013). This four-cluster solution was verified using K-means analysis with the Euclidean distances between Final Cluster Centers showing the distances between cluster 1 and cluster 2, cluster 1 and cluster 3, and cluster 1 and cluster 4 were 2.162, 2.003, and 1.696, respectively. K-means resulted in the following group sizes; seven (cluster 1), eight (cluster 2), five (cluster 3) and 20 (cluster 4). Cross-tabulation using the exact test was run to determine significance of variables within clusters. In line with the literature, Fisher's Exact Test was used instead of the Pearson Chi-Square test of significance given the modest sample size (Field, 2015). Column proportions within each category were compared using z-tests with Bonferroni correction applied (Eriksson et al., 2021).

## Results

### Demographics

Demographic data is provided in Table 2.

### Intimate Partner Violence: A Protoscript

#### Protoscript Development

The protoscript developed for this study drew from the original framework developed by Cornish (1994a) and those derived from both Leclerc et al. (2011) and Boxall et al. (2018). Initially drawing on a deductive approach, each intimate partner violence incident was analyzed to draw out key themes categorized using established script stages. Key words relevant to each of the pre-existing stages were identified and listed under the relevant stage. Similar words were then combined to reduce the number of themes to a manageable number. The pre-existing stages used in the study included historical preconditions (e.g., history of violence), situational preconditions (e.g., recent substance misuse or separation), location (private or public), and contact (originally referred to as target selection). Once the pre-existing stages were completed, inductive analysis was used to draw out more stages of the crime script. A coding book was developed under each stage of the script, with similar themes/terms combined (e.g., punching, kicking, and biting were combined under physical violence). Several iterations were developed and confirmed between the research team prior to development of the final themes. These themes were then mapped onto a revised protoscript framework to incorporate pre-existing and newly developed stages (discussed further below and shown in Figure 1). These added stages were useful for better reflecting the nature of offender/victim interaction and the sequence of events evident within each intimate partner incident.

#### Protoscript Stages

Original crime script stages incorporated preparation, entry, and preconditional stages to describe the lead up to crime commission (Cornish, 1994a). More recently, Boxall et al. (2018) revised preconditions to include historical and situational factors. This was to better acknowledge and incorporate the pattern-based nature of intimate partner violence. *Historical preconditions* aim to better understand the “baggage” carried by offenders and victims entering relationships and to better understand a course of conduct over time (Boxall et al., 2018). In this study, historical preconditions included the offender's history of domestic and family violence (within the same or past relationships), prior contraventions of domestic violence orders, frequency, previous strangulation, or history of controlling or jealous behavior, history of general violence, past separation, and/or a long-term history of problematic substance use (illicit drugs and/or alcohol) as identified in police narratives and DV-PAF indicators. *Situational preconditions* also aim to provide a greater understanding of the context for violence by identifying potential stressors (such as relationship breakdown), recent substance misuse (such as heavy drinking or drug taking just prior to the offence) or recent change in circumstances (such as loss of employment) (see, e.g., Boxall et al., 2018). This study categorized situational factors as those factors present anytime within the last week prior to the incident (recent relationship separation, escalating or ongoing conflict, recent stalking, and substance misuse just prior to the incident).

Incidents characterized by use of weapon, strangulation, or explicit threats to kill were included as *incident characteristics* to better identify incidents with high risk for lethality indicators (Dawson & Piscitelli, 2021; Domestic and Family Violence Death Review and Advisory Board, 2021). In the incidents studied, *contact*, like Cornish's (1994a) initiation stage, occurred when the victim and offender were already in one another's company just prior to the incident or because contact was initiated by the offender or the victim (in a small number of cases). variables used to describe *Escalation* (actualization) in the protoscript included jealousy, controlling behaviors (such as attempting to stop the victim from leaving the location), arguments over finances or general arguments. Offender actions and victim response were included as new stages of the script to reflect the distinct roles that offenders and victims play and how their actions may influence a continuation or cessation of violence. It also enables greater understanding of the methods used by a victim to de-escalate the situation, protect others who may be present or to protect themselves from further harm. *Offender actions* included physical and non-physical violence or property damage, with an additional variable “offender leaves” added to the *subsequent offender actions* stage. *Victim response* to the offender's behavior included doing as they were told (compliance) and calling for help (assistance), barring access to the location or attempting self-defense (challenge/protect). Given the data are drawn from police narratives and DV-PAF indicators, all calls for service involved some form of *intervention*, either through the arrival or involvement of police or a third party (such as a neighbor or family member prior to police involvement). *Offender reaction to intervention* included a continuation or further escalation of violence or departing the scene. *End of contact/conflict* occurred when police arrived on-scene and detained or removed the offender from the location, a police protection notice was issued or because one party left the location.

### Diversity Within Intimate Partner Violence Crime Scripts

Cluster analysis was used to examine whether the overall sample contained discrete types of groupings of script tracks. Fisher's Exact Test (two-tailed) was used to examine associations between cluster membership and script variables with significance set at 5%. Bonferroni's post hoc test was then completed to determine statistically significant differences between the four clusters (see Table 3).

**Table 3.** Cluster Analysis—Script Tracks.

Measures		Cluster 1 Escalating jealousy (%)	Cluster 2 Persistently possessive (%)	Cluster 3 Controlling victim agency (%)	Cluster 4 Enduring argument (%)	(χ^2^) *p*
*Historical precondition*	DFV	57.1	100.0	100.0	75.0	(4.95).138
	Violence	28.6	37.5	40.0	15.0	(2.86).364
	Substance misuse	28.6	25.0	20.0	20.0	(0.69).641
	Separation	0.0^a^	87.5^b^	60.0^a,b^	35.0^a,b^	(12.91).003
	Jealousy/control	14.3^a,b^	62.5^b^	20.0^a,b^	0.0^a^	(13.67).001
	Strangulation	0.0	37.5	20.0	5.0	(5.72).072
*Situational precondition*	Substance misuse	57.1	62.5	40.0	55.0	(0.81).932
	Ongoing conflict	85.7	62.5	80.0	50.0	(3.32).370
	Stalking	0.0^a,b^	37.5^b^	40.0^b^	0.0^a^	(10.32).005
*Incident characteristics*	Strangulation	28.6	12.5	20.0	20.0	(0.92).882
	Use of weapon	0.0	0.0	0.0	15.0	(1.87).712
	Explicit threats to kill	14.3	25.0	0.0	5.0	(3.07).262
	Sexual assault	0.0	0.0	20.0	0.0	(4.85).125
*Escalation*	Jealousy	57.1^a^	50.0^a^	20.0^a^	10.0^a^	(8.18).027
	Control	71.4^a,b^	87.5^b^	100.0^b^	20.0^a^	(17.69).000
	Financial	0.0	0.0	0.0	10.0	(1.51) 1.000
	Argument	0.0^a^	12.5^a,b^	0.0^a,b^	65.0^b^	(15.05).001
	Suicidal/attempts	0.0^a,b^	37.5^b^	0.0^a,b^	0.0^a^	(7.90).010
*Offender initial action*	Physical violence	28.6	62.5	20.0	45.0	(2.77).491
	Property damage	14.3	0.0	40.0	20.0	(3.36).341
*Victim initial response*	Challenge/protect/assist	71.4^a^	0.0^b^	100.0^a^	60.0^a^	(15.07).001
	Distance	14.3^a^	100.0^b^	0.0^a^	40.0^a^	(16.81).000
	Nil/comply	14.3	0.0	0.0	5.0	(2.12).551
*Offender subsequent action*	Physical violence	71.4	62.5	100.0	45.0	(5.35).143
	Property damage	14.3	37.5	40.0	25.0	(1.66).684
	Leave	14.3	0.0	0.0	15.0	(1.65).784
*Victim subsequent response*	Challenge/protect/assist	0.0	12.5	40.0	30.0	(3.74).296
	Distance	42.9	50.0	40.0	20.0	(3.33).345
	Nil/comply	14.3	0.0	20.0	10.0	(2.01).613
*Intervention*	Police called/intervene	14.3^a^	75.0^a,b^	60.0^a,b^	75.0^b^	(8.27).035
	Third party called/ intervene	85.7^a^	12.5^b^	0.0^b^	20.0^b^	(12.48).003
	Nil intervention	0.0	0.0	40.0	5.0	(5.35).107
*Offender action*	Continue/escalate	28.6	62.5	60.0	25.0	(4.69).194
	Leave	14.3	25.0	20.0	50.0	(3.69).286
	Stop violence	57.1^a^	0.0^a,b^	0.0^a,b^	5.0^b^	(9.88).006
*End of contact*	Police involvement	85.7^a,b,c^	100.0^c^	20.0^b^	100.0^a,c^	(15.87).000
	One party leaves	14.3^a,b,c^	0.0^c^	80.0^b^	0.0^a,c^	(15.87).000
% of total incidents		17.5	20.0	12.5	50.0	

*Note*. Each superscript letter (e.g., a) indicates a subset of group categories (i.e., clusters) whose proportions do not differ from one another at the .05 significance level. Percentages that are not statistically different share superscript letters, while percentages that are statistically different do not share superscript letters.

**Figure 2.**
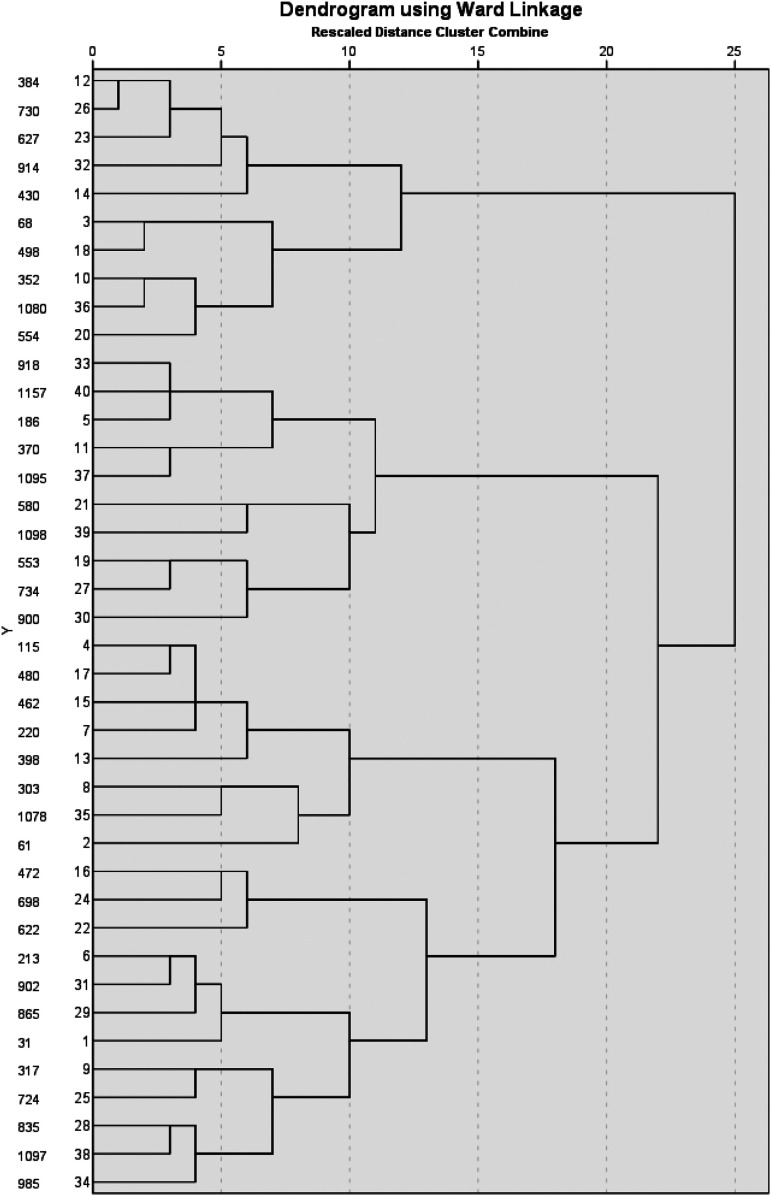
Four cluster analysis dendrogram.

Several variables differed significantly across clusters in varying combinations. These variables included a history of separation (*p* < .01), history of jealousy/control (*p* < .001), situational stalking (*p* < .05), escalating control (*p* < .001), escalating argument (*p* < .001), attempted suicide (*p* < .01), victim initial response to distance from the offender (*p* < .001), calling police (*p* < .05), third party intervention (*p* < .01), offender stops using violence (*p* < .01), end of contact due to police involvement (*p* < .001), and end of contact due to one party leaving the scene (*p* < .001) (Figure 2).

#### Cluster 1: Escalating Jealousy (*n* = 7)

Victims and offenders in cluster 1, which we label “Escalating Jealousy,” were aged between 18 years and over 50 years, with the largest portion of victims in cluster 1 aged less than 25 years (42.9%) and the largest portion of offenders aged between 35 and 49 years (42.9%) (see Table 1). Victims and offenders were classified as Australian (42.9%; 57.1%) or other (28.6%; 14.3%), respectively, with no couples identifying as Aboriginal and/or Torres Strait Islander people. Most couples in cluster 1 were co-residing (57.1%) at the time of the call for service, were in a current relationship (85.7%), and had children together (either biological or otherwise; 71.4%). All but two couples had length of relationship recorded with the majority indicating being in a relationship for more than 12 months at the time the call for service was responded to. Most of the victims (71.4%) reported feeling fearful or very fearful of the offender.

Historical preconditions evident within escalating jealousy included a history of intimate partner violence, general violence, and substance misuse, although these factors were common across all clusters and therefore not statistically associated with cluster membership (see Table 2). Couples in escalating jealousy had no prior history of separation, which differed significantly when compared to cluster 2 (87.5%). These relationships were characterized by escalating jealousy (thus the label), including accusations over infidelity. Relationships in escalating jealousy differed significantly to those recorded in enduring argument, with enduring argument relationships more likely to involve an escalating argument (65.0%) when compared to escalating jealousy. Victims in escalating jealousy were significantly more likely to seek help from a neighbor or family member during an incident (85.7%), with a statistically significant positive association across escalating jealousy and all other clusters. Offenders in escalating jealousy were more likely to stop using violence once intervention took place (57.1%), which was significantly different to those in enduring argument (5.0%).

#### Cluster 2: Persistently Possessive (*n* = 8)

Victims in cluster 2, which we label “Persistently Possessive,” were more likely to be aged between 25–34 years (75.0%) or 50 years and over (25.0%), with offender age ranging from between 25–34 years (50.0%), 35–49 years (25.0%) or 50 years and over (25.0%). As with cluster 1, victims in cluster 2 identified as Australian (50.0%), while offenders identified as Australian (37.5%) or other (12.5%). It should be noted that data on ethnicity was missing for half of the sample for persistently possessive. Relationship types differed within persistently possessive with couples either in a current relationship (37.5%), former relationship (25.0%), or a relationship categorized as on and off again (37.5%). Most couples in persistently possessive were co-residing (75.0%) at the time of the call for service, and just over one-third (37.5%) had children in the relationship. Many victims (87.5%) were fearful or very fearful.

Historical preconditions identified in persistently possessive included a history of separation (87.5%) that was statistically significant when compared to escalating jealousy, a history of strangulation (although this did not differ significantly across clusters), and a statistically significant association between a history of jealousy and control (62.5%) when compared to enduring argument. A statistically significant association with situational stalking (37.5%) was also found within persistently possessive when compared to enduring argument. Persistently possessive was also characterized by a statistically significant association with escalating jealousy and control (62.5%) when compared to enduring argument. The level of intimidation evident within persistently possessive was higher than all other clusters, with persistently possessive offenders more likely to follow the victim around the house, continue to verbally and/or physically abuse the victim and children when present, and use aggressive language such as forbidding a victim from saying or doing something. Offenders in persistently possessive were also more likely to make excessive phone calls, texts, and emails to the victim when compared to other clusters and were more likely to accuse their partner of cheating and needing to know the whereabouts of their partner. Offenders were also significantly more likely to attempt or threaten suicide in cluster 2 (37.5%) when compared to people in enduring argument. This may explain the strong association between victims in persistently possessive (100%) distancing themselves from the offender when compared to victims in all other clusters. Persistently possessive was significantly associated with ending due to police involvement (100%), particularly in comparison to controlling victim agency (20.0%).

#### Cluster 3: Controlling Victim Agency (*n* = 5)

Most victims in cluster 3, which we label “Controlling Victim Agency,” were aged between 25–34 years (80.0%), with offenders aged between 25–49 years (80.0%). Most victims (80.0%) and offenders (60.0%) identified as Australian, with the remainder identified as other (20%; 40.0%) respectively. As with escalating jealousy and persistently possessive, no couples in controlling victim agency identified as Aboriginal and/or Torres Strait Islander people. Just over half of the relationships in controlling victim agency were current at the time of the police call for service (60.0%) with the remainder classified as on and off again (40.0%). All the couples in controlling victim agency included children, with the majority co-residing at the time of the call for service (80.0%). Relationships in controlling victim agency varied in length with equal portions of less than 12 months (20.0%), between 12 months and five years (20.0%) and greater than five years (20.0%). Data was not available for 40% of the sample in controlling victim agency. A sizable portion of victims in controlling victim agency (60.0%) were recorded as being fearful or very fearful of the offender.

Historical preconditions evident in controlling victim agency included a history of intimate partner violence and separation, although these did not differ significantly across cluster membership. Situational preconditions included substance misuse, ongoing conflict, and stalking, although only stalking was statistically significant when compared to enduring argument. There was a negative association with jealousy in cluster 3 but a positive association with controlling behaviors, although this was not statistically different when compared to other clusters. Incidents in controlling victim agency were characterized by the offender turning up at the victim's residence, demanding to stay overnight, and attempting to sexually assault the victim. Victims in controlling victim agency were more likely to initially challenge the offender by way of refusing to allow them to stay or fighting back during a sexual assault when compared to victims in other clusters, although this was not statistically significant. In response, offenders escalated to physical violence that continued over an extended period with the victim then attempting to protect children and flee from the location, the offender taking keys, and blocking exits or taking hold of children to stop the victim leaving. End of contact/conflict in controlling victim agency was significantly associated with the offender leaving the location (80.0%) when compared to end of contact/conflict in persistently possessive.

#### Cluster 4: Enduring Argument (*n* = 20)

Cluster 4 was the largest cluster of the sample with half of all calls for service recorded. We label this cluster “Enduring Argument.” Victims within enduring argument were under 50 years of age, with 25.0% aged less than 25 years of age, 45.0% aged 25–34 years, and 30.0% aged 35–49 years. Offenders were spread across all age groups, with the largest portion aged 35–49 years. One quarter of the victims and offenders recorded in enduring argument identified as an Aboriginal and/or Torres Strait Islander person. Half of all victims were recorded as Australian along with 45.0% of offenders. Only a small percentage of offenders (5.0%) had other recorded ethnicity status. The remainder had no ethnicity recorded. Most people in enduring argument were in a current relationship (65.0%) at the time of the police call for service, with one quarter listed as former partners (25.0%) and a small number in an on and off again relationship (5.0%). Relationship length varied from less than 12 months (10.0%), 12 months to five years (35.0%) and greater than five years (10.0%). Victims in enduring argument (60.0%) were recorded as being fearful or very fearful of the offender.

As with all other clusters, enduring argument was characterized by historical precondition of a history of domestic and family violence, although this was not statistically significant. Unlike clusters one to three, incidents in enduring argument were significantly less likely to involve a history of jealousy or control. Situational preconditions including substance use at the time of the incident, ongoing conflict, and argument were evident in this cluster, with a strong positive association with the incident resulting from an escalating argument. These arguments started over money, substance misuse, ongoing relationship issues, unspecified reasons, or because the offender was angry. A victim's initial response was significantly associated with challenging the offender's behavior. There was also a positive association between police being called to these incidents and for the incident to end with police contact as opposed to the offender leaving the location.

## Discussion

This study was important for three reasons. Firstly, the protoscript developed aligns with past research (Boxall et al., 2018) and highlights that intimate partner violence follows similar stages of the crime commission process as other forms of interpersonal and general crimes. For example, Beauregard et al. (2007) identified stages of the hunting process of sex offenders that included preparation, entry to setting, committing the offence, and exiting the scene. Contact made with victim/offender, lead up to the act, committing the act, exit/end were also identified. Leclerc et al. (2011) found similar stages when examining child sex offenders with the protoscript including stages such as entry to setting, gaining trust, moving to location, committing the act, and avoiding disclosure strategies. Similar stages were evident in counterfeiting scripts with offenders first identifying opportunity for crime, preparation, committing the act and obtaining a benefit (Kennedy et al., 2018). Such information can provide opportunities to identify potential intervention points and offers insight into the types of prevention strategies that could be used by police and the service sector (Leclerc & Reynald, 2016; Leclerc et al., 2011) Secondly, this study has highlighted the diversity evident across intimate partner violence incidents, showing that while each track follows similar stages of the crime commission process outlined within the protoscript, offender actions and how victims respond vary. Thirdly, this study has identified the significance of specific variables and their association with script tracks.

### Key Findings

The intimate partner violence protoscript developed in this study supports the applicability of the original protoscript developed by Boxall et al. (2018) to other samples and suggests that intimate partner violence may be analyzed and understood in the same way as non-personal crimes (e.g., auto theft or burglary). At a broad level of analysis, this has the potential to influence policy and practice, particularly for first responders such as police. This protoscript also adds to the crime script literature by highlighting the methods used by victims to protect themselves or others during incidents and goes a little way in challenging stereotypes (Delgado-Alvarez & Sanchez-Prada, 2021) including around victim agency and their ability to effectively manage their safety (Nancarrow et al., 2020; Tolmie et al., 2018). This study includes additional information relevant to situational crime prevention by articulating the different methods used by offenders and victims, and the different intervention methods used.

Extensive prevalence literature shows intimate partner violence is commonplace (Australian Institute of Health and Welfare, 2019; Duke & Cunradi, 2011; World Health Organization, 2021a). Studies exploring intimate partner violence typologies often discuss violence in the context of generality and specialization (Myhill, 2015). Research has also identified substance misuse to be a problematic precursor to domestic violence (Gilchrist et al., 2019; Matias et al., 2020; Myhill & Hohl, 2019). In contrast, the current study found general violence to be prevalent across all typologies although analysis suggests its presence was not statistically significant across the clusters because it was present in each cluster. This was also true of a history of general violence and long-term substance misuse.

Historical preconditions involving a history of separation and jealous and controlling behaviors differed significantly between clusters. These were more likely to occur in incidents characterized by multiple significant variables such as historical strangulation and situational stalking (Bendlin & Sheridan, 2019; Love et al., 2020; Myhill & Hohl, 2019). Sexual violence was unique to the *controlling victim agency* script track at a statistically significant level and suggests first responders to these incidents should consider additional measures when identifying prevention and intervention strategies. It is important to note that while the controlling victim agency track was the only one to indicate sexual violence, data was derived from police administrative data. Given the dark figure of sexual crimes and significant barriers to reporting this form of violence it is probable that additional incidents involving sexual violence went unreported (Australian Institute of Health and Welfare, 2019; Buil-Gil et al., 2021; Griffin et al., 2021; Khan et al., 2018; Reich et al., 2021; Zinzow et al., 2021).

Risk literature consistently identifies escalating jealousy and control as significant factors in escalating violence and intimate partner homicide (H. Johnson et al., 2019; Myhill & Hohl, 2019; Saxton et al., 2020). Although this study found escalating jealousy and control to be prevalent in several clusters, it was not prevalent across all. An escalation in jealous and controlling behaviors was statistically significant, with a positive association between escalating jealousy evident within escalating jealousy and persistently possessive and controlling behaviors evident within escalating jealousy, persistently possessive and controlling victim agency. A negative association with jealousy was evident in controlling victim agency and enduring argument, while control was negatively associated with enduring argument. Unlike escalating jealousy, persistently possessive and controlling victim agency, a strong positive association was identified with enduring argument characterized by escalating arguments. A strong positive association with suicide threats/attempts was evident in persistently possessive but not within the other clusters. As persistently possessive is also characterized by long term and situational jealousy and control, it is likely offenders use these threats/attempts to control victims (Fontes, 2015; Victoria State Government, 2018).

Social understanding of how victims react to violence often fails to account for the ways victims attempt to protect themselves (Meyer, 2012). Instead, stereotypical views of “true victims” outline “socially accepted” strategies victims should engage in when seeking help (Nancarrow et al., 2020). This includes immediately reporting matters to police, cooperating with responding police, displaying physical injury, or acting subdued/quiet (Nancarrow et al., 2020). These stereotypes often ignore the complex decisions victims take when navigating violent relationships. In terms of victim responses to the initial violence by the offender, a statistically significant association was noted across clusters. For victims in cluster 2, there was a positive association with attempts to distance themselves from the offenders, whereas in the other clusters a positive association was noted in victims initially challenging the offender. Offenders were likely to escalate to violence after the victim's initial response in escalating jealousy and persistently possessive although this was not statistically significant. This finding, although based on only a small sample and not generalizable, counters perceptions that victims can just leave, and instead reveals the risk victims take when attempting to distance from an offender (Walsh, 2019; Webster et al., 2018). There was a positive association between police being called either by a party to the incident or a witness within persistently possessive and enduring argument, but a negative association within escalating jealousy and controlling victim agency. This aligns with literature exploring help-seeking behaviors that found help seeking increased as the severity of violence increased (Lelaurain et al., 2017). As would be expected, police involvement saw a positively and statistically significant association between end of contact/conflict due to police involvement within persistently possessive and enduring argument and a negative but significant association between police involvement in escalating jealousy and controlling victim agency. A strong positive association between the offender leaving the location after the incident was evident within controlling victim agency and a small but positive association within escalating jealousy.

### Implications for Situational Crime Prevention

It was evident from the clusters identified in this study that half of all intimate partner violence incidents were associated with ongoing or escalating arguments as opposed to jealous or controlling behaviors. This is an important distinction as research has shown that jealousy and controlling behaviors are high risk factors for future harm and lethality (Dobash et al., 2007; H. Johnson et al., 2019; Matias et al., 2020; Myhill & Hohl, 2019). This suggests that of all incidents attended by police, it is likely that 50% of incidents involve potentially high risk factors that must be identified and effectively addressed. For incidents such as those within the persistently possessive cluster, where several high risk factors including history of control, jealousy, separation, and ongoing jealous, controlling, and stalking behaviors are evident, more punitive responses may be required. The remainder of incidents that do not display controlling behaviors may require a different response, such as support to referral services, anger management, financial support, or other more generalist support options. Understanding diversity within intimate partner violence may support policy and procedural changes designed to better identify specific behaviors evident within incidents that police attend and provide guidance on the most appropriate action to take at the scene and following an intimate partner violence incident. Of course, this would require further investigation to establish guidelines outlining who would develop crime scripts, how these scripts would be used, and level of decision making required (for example Senior Constable, Sergeant level) among other decisions.

Emerging evidence suggests that the establishment of integrated responses to crime is becoming increasingly common. One of the main tasks of such teams is to provide holistic support to victims of interpersonal crimes such as intimate partner and/or sexual violence (Department of Child Safety Youth and Women, 2017, 2019). However, the current research suggests the need for an approach based on emerging evidence that considers the intersectional nature of interpersonal violence and the need for agencies to work together as opposed to in silos (Tsantefski et al., 2018). Integrated models that recognize the possible importance of heterogeneity when responding to domestic violence incidents are also beneficial as they can better identify and implement tailored situational crime prevention and intervention strategies. It is acknowledged that for integrated responses to work effectively, they must be founded in common practice (Mundy & Seuffert, 2021). The use of crime scripts in this context could ensure all agencies responsible for responding to intimate partner violence bring their own unique perspectives to identifying points of intervention within the script. They could then work together to implement these strategies to better protect victims and hold offenders accountable.

### Implications for Crime Script Analysis Research

Limited research to date has examined intimate partner violence using crime script analysis. What this study has shown is that like other forms of interpersonal and general crimes, intimate partner violence incidents follow the general crime commission process. It has also shown that within the overarching, distinct tracks emerge that highlight the diversity in intimate partner violence offending. The role of offender and victim are important to better understanding the sequence of events that occur within scripts, the ways in which victims within intimate partner violence attempt to protect themselves and others and how offenders react to this. This information will afford greater potential for prevention and intervention strategies. The use of cluster analysis gives strength to qualitative findings used to develop script tracks, highlighting the flexibility of crime script analysis for both qualitative and quantitative fields. This supports a discussion by Dehghaniri and Borrion (2021) in which they suggest that a change of direction from qualitative, thematic analysis of data will give way to a more structured quantitative approach to crime script analysis. Although qualitative analysis is important for understanding the intricacies evident within interpersonal crimes, this study shows that it is possible to produce crime scripts at varying levels of specificity in a more transparent manner using quantitative methods so that results may be reproduced in different samples.

### Limitations and Future Direction

Several limitations must be noted in the current study. Firstly, crime scripts were based on police administrative data that relies on an officer's perception of what information is relevant to capture. Secondly, within the Queensland context, police do not complete the DV-PAF report or police narrative on scene, instead recording information in their notebook and inputting the data into QPRIME upon returning to the station. If police are called to additional calls for service in between attending the intimate partner violence incident and completing the relevant paperwork, it is possible that some information may be unintentionally excluded from the report. Given the complexities of intimate partner violence, it is also possible that offenders and/or victims may not provide police with all the relevant information necessary to make informed decisions regarding the current and any previous incidents or levels of risk. Lastly, as research has highlighted, intimate partner violence is not a single incident but a series of incidents involving a pattern of abuse (Stark, 2012). While single incidents were used to develop the script tracks in the current study, it is possible that when viewed as a series of incidents, patterns of behavior may change.

As emerging research notes (see Fontes, 2015; McMahon & McGorrery, 2020; Myhill, 2015), intimate partner violence is rarely a single incident, but a pattern of ongoing abuse. This single-based view of intimate partner violence has led to current criticism of the policing model that oftentimes fails to acknowledge the ongoing pattern of abuse experienced by victims (Stark, 2012). This type of response also fails to address the underlying causes of violence, fails to accurately identify the primary victim, can potentially impact victim safety, and limits the options available for prevention. Future research must examine intimate partner violence by analyzing a series of incidents involving the same offender/victim as well as offenders with multiple victims to determine whether similar patterns of behavior are used over time. By understanding general patterns of offending across different types of intimate partner violence scripts, police and service providers will be able to better identify underlying factors that lead to violence and develop more effective prevention and interventions tailored to the situation. Finally, the study used a small sample of cases (*n* = 40) from a single Australian jurisdiction. As such, generalizability is not able to be established. It is suggested further research is conducted, including expanding the sample to include a larger number of cases from multiple jurisdictions. Despite the limitations, this study has shown that conceptualizing intimate partner violence in a way that is accessible to non-specialists may be beneficial. By raising awareness and shifting current understandings of the dynamics of intimate partner violence, it may be possible to draw on the methods explored in this study to better inform responses. This could be achieved by developing evidence-based, readily accessible tools to assist frontline officers in identifying intimate partner typologies that can then be used to explore tailored options for intervention.
